# 3D Wavelet Subbands Mixing for Image Denoising

**DOI:** 10.1155/2008/590183

**Published:** 2008-04-08

**Authors:** Pierrick Coupé, Pierre Hellier, Sylvain Prima, Charles Kervrann, Christian Barillot

**Affiliations:** ^1^University of Rennes I, CNRS UMR 6074, IRISA, F-35042 Rennes, France; ^2^VisAGeS U746 Unit/Project, INRIA, IRISA, F-35042 Rennes, France; ^3^VisAGeS U746 Unit/Project, IRISA, INSERM, F-35042 Rennes, France; ^4^UR341 Mathematiques et Informatique Appliquées, INRA, F-78352 Jouy en Josas Cedex, France; ^5^VISTA Project-Team, IRISA, INRIA, F-35042 Rennes, France

## Abstract

A critical issue in image restoration is the problem of noise removal while keeping the integrity of relevant image information. The method proposed in this paper is a fully automatic 3D blockwise version of the nonlocal (NL) means filter with wavelet subbands mixing. The proposed wavelet subbands mixing is based on a multiresolution approach for improving the quality of image denoising filter. Quantitative validation was carried out on synthetic datasets generated with the BrainWeb simulator. The results show that our NL-means filter with wavelet subbands mixing outperforms the classical implementation of the NL-means filter in terms of denoising quality and computation time. Comparison with wellestablished methods, such as nonlinear diffusion filter and total variation minimization, shows that the proposed NL-means filter produces better denoising results. Finally, qualitative results on real data are presented.

## 1. INTRODUCTION

Image denoising can be considered as a component of processing or as a process itself. In the first case, the image denoising is used to improve the accuracy of various image processing algorithms such as registration or segmentation. Then, the quality of the artifact correction influences performance of the procedure. In the second case, the noise removal aims at improving the image quality for visual inspection. The preservation of relevant image information is important, especially in a medical context.

This paper focuses on a new denoising method firstly introduced by Buades et al. [1] for 2D image denoising: the nonlocal (NL) means filter. We propose, to improve this filter with an automatic tuning of the filtering parameter, a blockwise implementation and a mixing of wavelet su-bands based on the approach proposed in [2]. These contributions lead to a fully-automated method and overcome the main limitation of the classical NL-means: the computational burden.

Section 2 presents related works. Section 3 presents the proposed method with details about our contributions. Section 4 shows the impact of our adaptations compared to different implementations of the NL-means filter and proposes a comparison with well-established methods. The validation experiments are performed on a phantom dataset in a quantitative way. Finally, Section 5 shows results on real data.

## 2. RELATED WORKS

Many methods for image denoising have been suggested in the literature, and a complete review of them can be found in [1]. Methods for image restoration aim at preserving the image details and local features while removing the undesirable noise. In many approaches, an initial image is progressively approximated by filtered versions which are smoother or simpler in some sense. Total variation (TV) minimization [3], nonlinear diffusion [4–6], mode filters [7], or regularization methods [3, 8] are among the methods of choice for noise removal. Most of these methods are based on a weighted average of the gray values of the pixels in a spatial neighborhood [9, 10]. One of the earliest examples of such filters has been proposed by Lee [11]. An evolution of this approach has been presented by Tomasi and Manduchi [9] who devised the bilateral filter which includes both a spatial and an intensity neighborhood.

Recently, the relationships between bilateral filtering and local mode filtering [7], local *M*-estimators [12], and nonlinear diffusion [13] have been established. In the context of statistical methods, the bridge between the Bayesian estimators applied on a Gibbs distribution, resulting with a penalty functional [14] and averaging methods for smoothing, has also been described in [10]. Finally, statistical averaging schemes enhanced via incorporating a variable spatial neighborhood scheme have been proposed in [15–17].

All these methods aim at removing noise while preserving relevant image information. The tradeoff between noise removal and image preservation is performed by tuning the filter parameters, which is not an easy task in practice. In this paper, we propose to overcome this problem with a 3D subbands wavelet mixing. As in [2], we have chosen to combine a multiresolution approach with the NL-means filter [1], which has recently shown very promising results.

Recently introduced by Buades et al. [1], the NL-means filter proposes a new approach for the denoising problem. Contrary to most denoising methods based on a local recovery paradigm, the NL-means filter is based on the idea that any periodic, textured, or natural image has redundancy, and that any voxel of the image has similar voxels that are not necessarily located in a spatial neighborhood. This new *nonlocal recovery paradigm* allows to improve the two most desired properties of a denoising algorithm: edge preservation and noise removal.

## 3. METHODS

**Figure 1:**
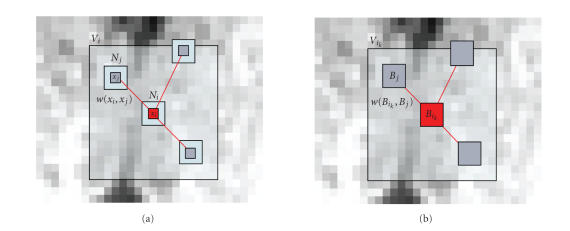
(a) Usual voxelwise NL-means filter: 2D illustration of the NL-means principle. The restored value of voxel *x*_*i*_ (in red) is the weighted average of all intensities of voxels *x*_*j*_ in the search volume *V*_*i*_, based on the similarity of their intensity neighborhoods **u**(*N*_*i*_) and **u**(*N*_*j*_). In this example, we set *d* = 1 and *M* = 8. (b) Blockwise NL-means filter: 2D illustration of the blockwise NL-means principle. The restored value of the block *B*_*i*_*k*__ is the weighted average of all the blocks *B*_*j*_ in the search volume *V*_*i*_*k*__. In this example, we set *α* = 1 and *M* = 8.

**Figure 2:**
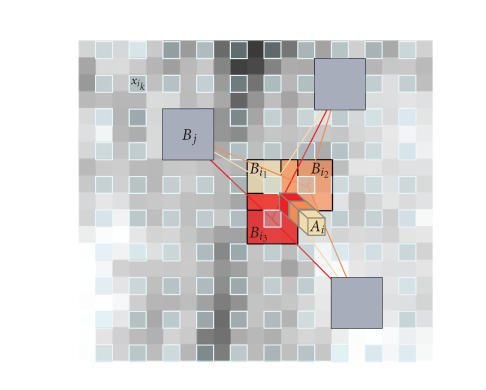
Blockwise NL-means filter. For each block *B*_*i*_*k*__ centered on voxel *x*_*i*_*k*__, an NL-means-like restoration is performed from blocks *B*_*j*_. In this way, for a voxel *x*_*i*_ included in several blocks, several estimations are obtained. The restored value of voxel *x*_*i*_ is the average of the different estimations stored in vector **A**_*i*_. In this example, *α* = 1, *n* = 2, and |**A**_*i*_| = 3..

In this section, we introduce the following notations:

- *u* : Ω^3^ → ℝ is the image, where Ω^3^ represents the image grid, considered as cubic for the sake of simplicity and without loss of generality (|Ω^3^| = *N* ^3^);
- for the original voxelwise NL-means approach,
  - *u*(*x*_*i*_) is the intensity observed at voxel *x*_*i*_,
  - *V*_*i*_ is the cubic search volume centered on voxel *x*_*i*_ of size |*V*_*i*_| = (2*M* + 1)^3^, *M* ∈ ℕ ,
  - *N*_*i*_ is the cubic local neighborhood of *x*_*i*_ of size |*N*_*i*_| = (2*d* + 1)^3^, *d* ∈ ℕ ,
  - **u**(*N*_*i*_) = (*u* ^(1)^ (*N*_*i*_),…, *u* ^(|*N*_*i*_ |)^(*N*_*i*_))^*T*^ is the vector containing the intensities of *N*_*i*_ (that we term “patch” in the following),
  - *NL*(*u*)(*x*_*i*_) is the restored value of voxel *x*_*i*_,
  - *w*(*x*_*i*_, *x*_*j*_) is the weight of voxel *x*_*j*_ when restoring *u*(*x*_*i*_) (see Figure 1(a));
- for the blockwise NL-means approach,
  - *B*_*i*_ is the block centered on *x*_*i*_ of size |*B*_*i*_| = (2*α* + 1)^3^, *α* ∈ ℕ,
  - **u**(*B*_*i*_) is the vector containing the intensities of the block *B*_*i*_,
  - **NL**(*u*)(*B*_*i*_) is the vector containing the restored value of *B*_*i*_,
  - *w*(*B*_*i*_, *B*_*j*_) is the weight of block *B*_*j*_ when restoring the block **u**(*B*_*i*_) (see Figure 1(b)),
  - the blocks *B*_*i*_*k*__ are centered on voxels *x*_*i*_*k*__ which represent a subset of the image voxels, equally regularly distributed over Ω^3^ (see Figure 2),
  - *n* represents the distance between the centers of the blocks *B*_*i*_*k*__ (see Figure 2).

### 3.1. The nonlocal means filter

In the classical formulation of the NL means filter [1], the restored intensity NL(*u*) (*x*_*i*_) of the voxel *x*_*i*_, is a weighted average of the voxels intensities *u*(*x*_*i*_) in the “search volume” *V*_*i*_ of size (2*M* + 1)^3^:

(1) $$\text{NL}(u)(x_{i})=\underset{x_{j}\in V_{i}}{\sum }w(x_{i},x_{j})u(x_{j}),$$

where *w*(*x*_*i*_, *x*_*j*_) is the weight assigned to value *u*(*x*_*j*_) to restore voxel *x*_*i*_. More precisely, the weight evaluates the similarity between the intensity of the local neighborhoods *N*_*i*_ and *N*_*j*_ centered on voxels *x*_*i*_ and *x*_*j*_, such that *w*(*x*_*i*_, *x*_*j*_) ∈ [0, 1] and ∑_*x*_*j*_∈*V*_i**__ *w*(*x*_*i*_, *x*_*j*_) = 1 (cf., Figure 1, Left).

For each voxel *x*_*j*_ in *V*_*i*_, the computation of the weight is based on the Euclidean distance between patches **u**(*N*_*j*_) and **u**(*N*_*i*_), defined as

(2) $$w(x_{i},x_{j})=\frac{1}{Z_{i}}e^{-∥u(N_{i})-u(N_{j}){∥}_{2}^{2}/h^{2}},$$

where *Z*_*i*_ is a normalization constant ensuring that ∑_*j*_ *w*(*x*_*i*_, *x*_*j*_) = 1, and *h* acts as a filtering parameter controlling the decay of the exponential function.

#### 3.1.1. Automatic tuning of the filtering parameter *h*

As explained in the introduction, denoising is usually the first step of complex image processing procedures. The number and the dimensions of the data to process being continually increasing, each step of the procedures needs to be as automatic as possible. In this section, we propose an automatic tuning of the filtering parameter *h*

First, it has been shown that the optimal smoothing parameter *h* is proportional to the standard deviation of the noise *σ* [1]. Second, if we want the filter independent of the neighborhood size, the optimal *h* must depend on |*N*_*i*_| (see, (2)). Thus, the automatic tuning of the filtering parameter *h* amounts to determining the relationship *h* ^2^ = *f* (*σ* ^2^, |*N*_*i*_|, *β*), where *β* is a constant.

Firstly, the standard deviation of the noise *σ* needs to be estimated. In case of an additive white Gaussian noise, this estimation can be based on pseudoresiduals $\epsilon_{i}$ as defined in [18, 19]. For each voxel *x*_*i*_ of the volume Ω^3^ , let us define

(3) $$\epsilon_{i}=\sqrt{\frac{6}{7}}\left(u(x_{i})-\frac{1}{6}\underset{x_{j}\in P_{i}}{\sum }u(x_{j})\right),$$

*P*_*i*_ being the 6-neighborhood at voxel *x*_*i*_ and the constant $\sqrt{6/7}$ is used to ensure that E[ϵi2]=σ^2 in the homogeneous areas. Thus, the standard deviation of noise $\hat{\sigma }$ is computed as

(4) σ^2=1|Ω3|∑i∈Ω3ϵi2.

Then, in order to make the filter independent of |*N*_*i*_|, we used the Euclidean distance || ⋅ ||^2^_2_ normalized by the number of elements:

(5) $$\frac{1}{\left|N_{i}\right|}∥u(N_{i})-u(N_{j}){∥}_{2}^{2}=\frac{1}{\left|N_{i}\right|}\overset{\left|N_{i}\right|}{\underset{p=1}{\sum }}{\left(u^{\left(p\right)}(N_{i})-u^{\left(p\right)}(N_{j})\right)}^{2}.$$

Based on the fact that, in the case of Gaussian noise and with normalized L2-norm, the optimal denoising is obtained for *h* ^2^ = 2*σ* ^2^ [20], (2) can be written as

(6) w(xi,xj)=1Zie−∥u(Ni)−u(Nj)∥22/2βσ^2|Ni|,

where only the adjusting constant *β* needs to be manually tuned. If our estimation $\hat{\sigma }$ of the standard deviation of the noise *σ* is correct, *β* should be close to 1. The optimal choice for *β* will be discussed later.

#### 3.1.2. Blockwise implementation

The main problem of the NL-means filter is being its computational time, a blockwise approach can be used to decrease the algorithmic complexity. Indeed, instead of denoising the image at a voxel level, entire blocks are directly restored.

A blockwise implementation of the NL-means filter consists in (a) dividing the volume into blocks with overlapping supports, (b) performing NL-means-like restoration of these blocks, and (c) restoring the voxels values based on the restored values of the blocks they belong to, as follows.

1. A partition of the volume Ω^3^ into overlapping blocks *B*_*i*_*k*__ of size (2*α* + 1)^3^ is performed, such as Ω^3^ = ∪_*k*_ *B*_*i*_*k*__ , under the constraint that each block *B*_*i*_*k*__ intersects with at least one other block of the partition. These blocks are centered on voxels *x*_*i*_*k*__ which constitute a subset of Ω^3^. The voxels *x*_*i*_*k*__ are equally distributed at positions *i*_*k*_ = (*k*_1_ *n*,*k*_2_ *n*, *k*_3_ *n*), (*k*_1_, *k*_2_, *k*_3_) ∈ ℕ^3^, where *n* represents the distance between the centers of *B*_*i*_*k*__ . To ensure a global continuity in the denoised image, the overlapping support of blocks is nonempty: 2*α* ≥ *n*.
2. For each block *B*_*i*_*k*__ , an NL-means-like restoration is performed as follows:
  (7) NL(u)(Bik)=∑Bj∈Vikw(Bik,Bj)u(Bj),with w(Bik,Bj)=1Zike−∥u(Bik)−u(Bj)∥22/2βσ^2|Ni|,
  where *Z*_*i*_*k*__ is a normalization constant ensuring that ∑_*j*_ *w*(*B*_*i*_*k*__ , *B*_*j*_) = 1 (see Figure 1, Right).
3. For a voxel *x*_*i*_ included in several blocks *B*_*i*_*k*__ , several estimations of the restored intensity NL(*u*)(*x*_*i*_) are obtained in different **NL**(*u*)(*B*_*i*_*k*__ ). The estimations given by different **NL**(*u*)(*B*_*i*_*k*__ ) for a voxel *x*_*i*_ are stored in a vector **A**_*i*_. The final restored intensity of voxel *x*_*i*_ is then defined as
  (8) $$\text{NL}(u)(x_{i})=\frac{1}{\left|A_{\text{i}}\right|}\underset{p\in A_{\text{i}}}{\sum }A_{i}(p),$$
  where **A**_*i*_(*p*) denotes the *p*th element of the vector **A**_*i*_.

The main advantage of this approach is to significantly reduce the complexity of the algorithm. Indeed, for a volume Ω^3^ of size *N* ^3^, the global complexity is *𝒪*((2*α* + 1)^3^ (2*M* + 1)^3^ ((*N* − *n*)/*n*)^3^). For instance, with *n* = 2, the complexity is divided by a factor 8.

#### 3.1.3. Block selection

In [21–23], the authors have shown that neglecting the voxels/blocks with small weights (i.e., the most dissimilar patches to the current one) speeds up the filter and significantly improves the denoising results. Indeed, the selection of the most similar patches **u**(*B*_*j*_) to the current patch **u**(*B*_*i*_) to compute **NL**(*u*)(*B*_*i*_) can be viewed as a spatially adaptation of the patch dictionaries. As in [21–23], the preselection of blocks in *V*_*i*_ is based on the mean and the variance of **u**(*B*_*i*_) and **u**(*B*_*j*_). The selection tests are given by

(9) w(Bik,Bj)={1Zike−∥u(Bik)−u(Bj)∥22/2βσ^2|Ni|  ⁢  if  μ1<u(Bik)¯u(Bj)¯<1μ1,   ⁢  σ12<Var(u(Bik))Var(u(Bj))<1σ12,0  otherwise,

where $\bar{u(B_{i_{k}})}$ and Var(**u**(*B*_*i*_*k*__ )) represent, respectively, the mean and the variance of the intensity function for the block *B*_*i*_*k*__ centered on the voxel *x*_*i*_*k*__ . The new parameters 0 < *μ*_1_ < 1 and 0 < *σ*_1_ < 1 control the level of rejection related to tests. When *μ*_1_ and *σ*_1_ are close to 0, there is almost no selection and the number of patches taken into account increases: thus the denoised image becomes smoother. The filter is equivalent to the classical NL-means and the computation time increases. When *μ*_1_ and *σ*_1_are close to 1, the selection is more severe and the number of patches taken into account decreases: the denoised image is less smoothed and the computation time decreases. This kind of selection tends to better enhance the contrast. In practice, *μ*_1_ and *σ*_1_ were chosen as in [21, 22]: *μ*_1_ = 0.95 and *σ*_1_ = 0.5.

### 3.2. Wavelet subbands mixing

#### 3.2.1. Hybrid approaches

Recently, hybrid approaches coupling the NL-means filter and a wavelet decomposition have been proposed [2, 24, 25]. In [24], a wavelet-based denoising of blocks is performed before the computation of the nonlocal means. The NL-means filter is performed with denoised version of blocks in order to improve the denoising result. In [25], the NL-means filter is applied directly on wavelet coefficients in transform domain. This approach allows a direct denoising of compressed images (such as JPEG2000) and a reduction of computational time since smaller images are processed. In [2], a multiresolution framework is proposed to adaptively combine the result of denoising algorithms at different space-frequency resolutions. This idea relies on the fact that a set of filtering parameters is not optimal over all the space-frequency resolutions. Thus, by combining to the transform domain the results obtained with different sets of filtering parameters, the denoising is expected to be improved.

#### 3.2.2. Overall processing

In order to improve the denoising result of the NL-means filter, we propose a multiresolution framework similar to [2] to implicitly adapt the filtering parameters (*h*, |*B*_*i*_|) over the different space-frequency resolutions of the image. This adaptation is based on the fact that the size of the patches impacts the denoising properties of the NL-means filter. Indeed, the weight given to a block depends on its similarity with the block under consideration, but the similarity between the blocks depends on their sizes. Thus, given the size of the blocks, removal or preservation of image components can be favored.

In the transform domain, the main features of the image correspond to low-frequency information while finer details and noise are associated to high frequencies. Nonetheless, noise is not a pure high-frequency component in most images. Noise is spanned over a certain range of frequencies in the image with mainly middle and high components [2].

**Figure 3:**
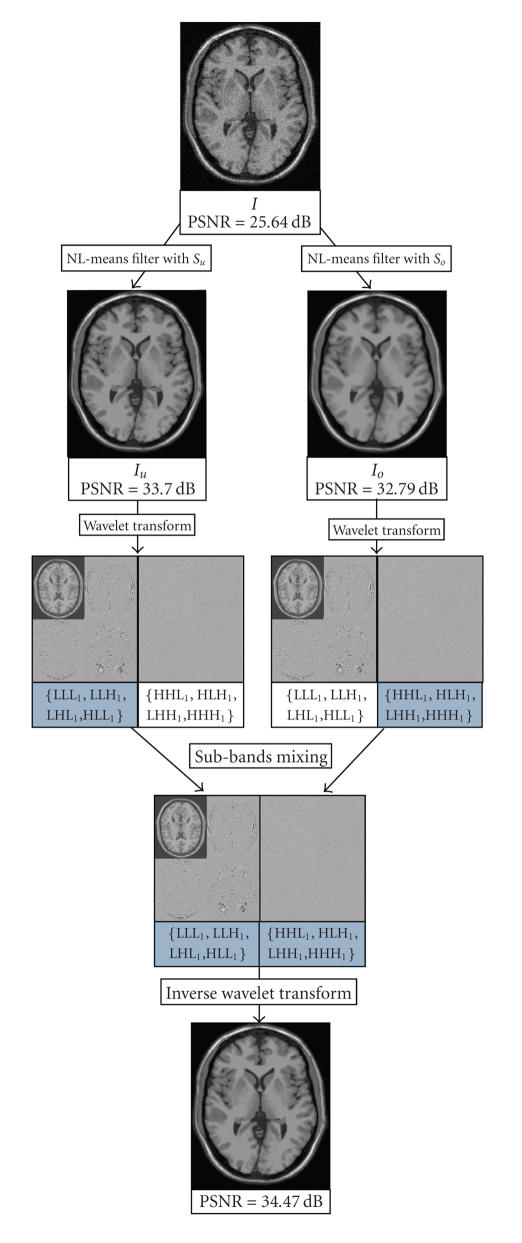
Workflow. First, the noisy image *I* is denoised with two sets of filtering parameters *S*_*u*_ and *S*_*o*_. Then, *I*_*u*_ and *I*_*o*_ are decomposed into low- and high-frequency subbands by 3D DWT. The four lowest frequency subbands of *I*_*u*_ (i.e., LLL_1_, LLH_1_, LHL_1_, and HLL_1_) are mixed with the four highest-frequency subbands of *I*_*o*_ (i.e., LHH_1_, HLH_1_, HHL_1_, and HHH_1_). Finally, the result image is obtained by inverse 3D DWT of the selected subbands.

In NL-means-based restoration, large blocks and setting *β* = 1 efficiently remove all frequencies of noise but tend to spoil the main features of the image, whereas small blocks and low smoothing parameter (*β* = 0.5) tend to better preserve the image components but cannot completely remove all frequencies of noise. As a consequence, we propose the following workflow (see Figure 3).

- Denoising of the original image *I* using two sets of filtering parameters: one adapted to the noise components removal (i.e., large blocks and *β* = 1) and the other adapted to the image features preservation (i.e., small blocks and *β* = 0.5). This yields two images *I*_*o*_ and *I*_*u*_. In *I*_*o*_, the noise is efficiently removed and, conversely, in *I*_*u*_, the image features are preserved.
- Decomposing *I*_*o*_ and *I*_*u*_ into low- and high-frequency subbands. The first level decomposition of the images is performed with a 3D discrete wavelet transform (DWT).
- Mixing the highest-frequency subbands of *I*_*o*_ and the lowest frequency subbands of *I*_*u*_.
- Reconstructing the final image by an inverse 3D DWT from the combination of the selected high and low frequencies.

In this paper, we propose an implementation of this approach using our optimized blockwise NL-means filter and the 3D DWT Daubechies-8 basis. The latter is implemented in Qccpack (http://qccpack.sourceforge.net) in the form of dyadic subband pyramids. This DWT is widely used in image compression due to its robustness and efficiency.

#### 3.2.3. Selection of wavelet subbands

Once the original image *I* has been denoised using two sets of filtering parameters, a 3D DWT at the first level is performed on both *I*_*o*_ and *I*_*o*_ images. For each image, eight subbands are obtained: LLL_1_, LLH_1_, LHL_1_, HLL_1_, LHH_1_, HLH_1_, HHL_1_, and HHH_1_.

- In the eight wavelet subbands obtained with *I*_*o*_, the frequencies corresponding to noise are efficiently removed from the high frequencies whereas the low frequencies associated to the main features are spoiled.
- In the eight wavelet subbands obtained with *I*_*u*_, the low frequencies associated to main features are efficiently preserved whereas residual frequencies corresponding to noise are present in high frequencies.

Thus, we select the highest frequencies of *I*_*o*_ (i.e., LHH_1_, HLH_1_, HHL_1_, and HHH_1_) and the lowest frequencies of *I*_*u*_ (i.e., LLL_1_, LLH_1_, LHL_1_, and HLL_1_). Then, the 4 lowest subbands of *I*_*u*_ are combined with the 4 highest subbands of *I*_*o*_. Finally, an inverse 3D DWT is performed on these 8 selected subbands to obtain the final denoised image (see Figure 3).

In [21, 22], the optimal parameters for 3D MRI have been estimated as *α* = 1, *M* = 5, *μ*_1_ = 0.95, and *σ*_1_ = 0.5. In our experiments, the two sets of parameters used to obtain *I*_*u*_ and *I*_*o*_ were *S*_*u*_ = (*α*_*u*_, *M*_*W*_, *β*_*u*_) = (1, 3, 0, 5) and *S*_*o*_ = (*α*_*o*_, *M*_*W*_, *β*_*o*_) = (2, 3, 1). Compared to [21, 22], the size of “search volume” was reduced to decrease the computational time. Several sets of parameters have been tested, the mentioned numerical values are satisfying to balance the denoising performance (high PSNR values) and computational burden. Finally, to decrease the computational time, this workflow is parallelized and each version is computed on different CPUs or cores (see Figure 3).

## 4. VALIDATION ON A PHANTOM DATA SET

### 4.1. Materials

In order to evaluate the performance of the different variants of the NL-means filter on 3D MR images, tests were performed on the BrainWeb database [26]. Several images were simulated to validate the performance of the denoising on various images: (a) T1-w phantom MRI for 4 levels of noise 3%, 9%, 15%, and 21% and (b) T2-w phantom MRI with multiple sclerosis (MS) lesions for 4 levels of noise 3%, 9%, 15%, and 21%. A white Gaussian noise was added, and the notations of BrainWeb are used: a noise of 3% is equivalent to *𝒩* (0, *ν*(3/100)), where *ν* is the value of the highest voxel intensity of the phantom (150 for T1-w and 250 for T2-w).

### 4.2. Comparison with different NL-means filters

In the following, let us define the following.

- *NL-means*: standard voxelwise implementation with automatic tuning of the filtering parameter *h* (*β* = 1) [1].
- *Optimized NL-means*: voxelwise implementation with automatic tuning of the filtering parameter *h* (*β* = 1) and voxels selection presented in [21].
- *Optimized blockwise NL-means*: (This filter can be freely tested at http://www.irisa.fr/visages/bench marks) blockwise implementation with automatic tuning of the filtering parameter *h* (*β* = 1) and blocks selection presented in [22].
- *Optimized blockwise NL-means with wavelet mixing*: proposed filter based on a blockwise implementation, an automatic tuning of the filtering parameter *h* (*β* = 1), a block selection, and a wavelet subbands mixing.

The selected filtering parameters for the different implementations were as follows.

- For the *NL-means* and *optimized NL-means* filters, the parameters are those used in [21]: *d* = 1, *β* = 1, *M* = 5, *μ*_1_ = 0.95 and *σ* ^2^_1_ = 0.5.
- Concerning the *optimized blockwise NL-means* filter, the sets of parameters are those used in [22]: *n* = 2, *α* = 1, *β* = 1, *M* = 5, *μ*_1_ = 0.95 and *σ* ^2^_1_ = 0.5.
- Finally, for the *optimized blockwise NL-means with wavelet mixing* filter the parameter are the following: *n* = 2, *S*_*u*_ = (*α*_*u*_, *M*_*W*_, *β*_*u*_) = (1, 3, 0.5), *S*_*o*_ = (*α*_*o*_, *M*_*W*_, *β*_*o*_) = (2, 3, 1), *μ*_1_ = 0.95, and *σ* ^2^_1_ = 0.5.

For 8-bit encoded images, the PSNR is defined as follows:

(10) $$\text{PSNR}=20\,\;\log_{10}\frac{255}{\text{RMSE}},$$

where RMSE denotes the root mean square error estimated between the ground truth and the denoised image. For the sake of clarity, the PSNR values are estimated only in the region of interest (cerebral tissues) obtained by removing the background (i.e., the label 0 of the discrete model in Brainweb).

**Figure 4:**
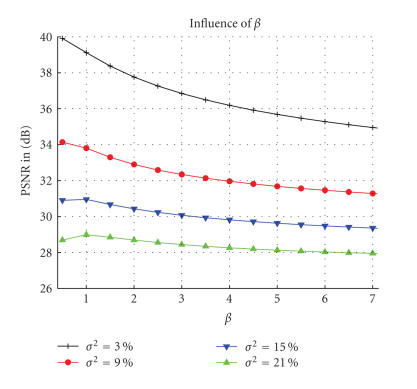
Influence of the filtering parameter $2\beta {\hat{\sigma }}^{2}$ on the PSNR according to *β* and for several levels of noise. These results are obtained with the *optimized blockwise NL-means* filter on the T1-w phantom MRI and account for the error in the estimation of *σ*.

Firstly, we have experimentally verified that the optimal denoising is obtained for *β* ≈ 1 for high levels of noise and *β* ≈ 0.5 for low levels of noise. These results account for the error in the estimation of *σ* (${\hat{\sigma }}^{2}=3.42\%$ at 3%, ${\hat{\sigma }}^{2}=7.93\%$ at 9%, ${\hat{\sigma }}^{2}=12.72\%$ at 15%, and ${\hat{\sigma }}^{2}=17.44\%$ at 21%) (see Figure 4). The parameter *β* was fixed to 1 for all the experiments.

#### 4.2.1. Quantitative results

**Figure 5:**
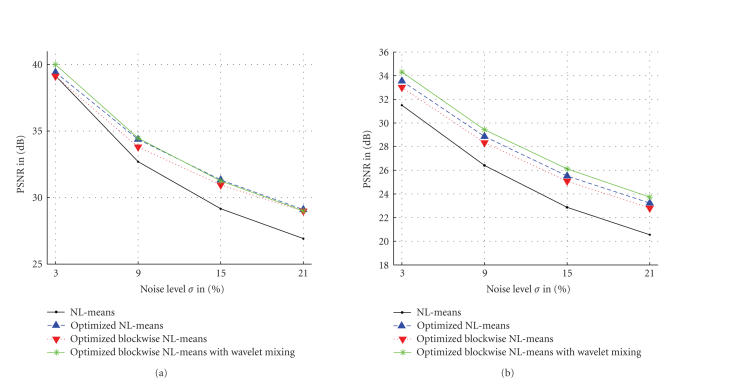
Comparison of the different NL-means filters on T1-w phantom MRI and T2-w phantom MRI with MS.

Table 1 shows that the blockwise approach of the NL-means filter, with and without voxels selection (see (9)), allows to drastically reduce the computational time. With a distance between the block centers corresponding to *n* = 2, the blockwise approach divides the timings by a factor superior to 5 (see Table 1). However, the computational time reduction is balanced with a slight decrease of the PSNR (see Figure 5) compared to the *optimized NL-means* filter presented in [21]. Our *optimized blockwise NL-means with wavelet mixing* allows to compensate this slight decrease of the PSNR and to divide the computational by a factor 4 compared to the *optimized NL-means* filter.

#### 4.2.2. Visual assessment

**Figure 6:**
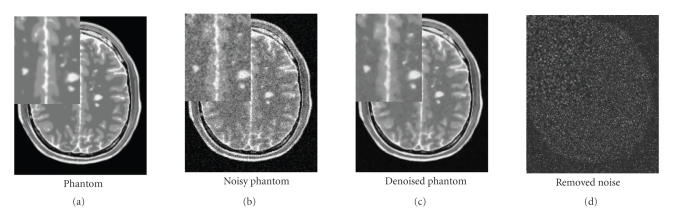
Fully automatic restoration obtained with the optimized blockwise NL-means with wavelet mixing filter in 3 minutes on a DualCore Intel(R) Pentium(R) D CPU 3.40 GHz. The image is a T2-w phantom MRI with MS of 181 × 217 × 181 voxels and 9% of noise.

**Figure 7:**
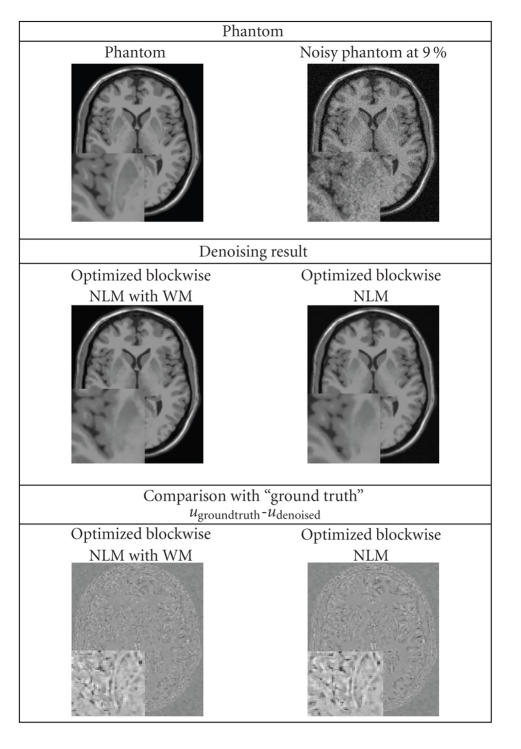
Top. Phantom and phantom noisy with 9%. Middle. The denoising result obtained with the *optimized blockwise NLM with WM* filter and the *optimized blockwise NLM* filter. Bottom. The image of difference between the phantom and the denoising result (i.e., *u*_groundtruth_-*u*_denoised_). The contrast of the zooms have been artificially increased. Visually, less structures have been removed with the *optimized blockwise NLM with WM* filter.

Visually, the proposed method combines the most important attributes of a denoising algorithm: edge preservation and noise removal. Figure 6 shows that our filter removes noise while keeping the integrity of MS lesions (i.e., no structure appears in the removed noise). Figure 7 focuses on the differences between the *optimized blockwise NLM* and the *optimized blockwise NLM with WM* filters. The denoising result obtained with the *optimized blockwise NLM with WM* filter visually preserves the edges better than the *optimized blockwise NLM* filter. This is also confirmed by visual inspection of the comparison with the “ground truth”. The images of difference between the phantom and the denoised image (see bottom of Figure 7) show that less structures have been removed with the *optimized blockwise NLM with WM* filter. Thus, the multiresolution approach allows to better preserve the edges and to enhance the contrast between tissues.

### 4.3. Comparison with other methods

In this section, we compare the proposed method with two of the most used approaches in MRI domain: the nonlinear diffusion (NLD) filter *r* [4] and the total variation (TV) minimization [3]. The main difficulty to achieve this comparison is related to the tuning of smoothing parameters in order to obtain the best results for NLD filter and TV minimization scheme. After quantifying the parameter space, we exhaustively tested all possible parameters within a certain range. This allows us to obtain the best possible results for the NLD filter and the TV minimization.

For the *optimized blockwise NLM with WM*, the same set of parameters *S*_*u*_ = (*α*_*u*_, *M*_*W*_, *β*_*u*_) = (1, 3, 0.5) and *S*_*o*_ = (*α*_*o*_, *M*_*W*_, *β*_*o*_) = (2, 3, 1) are used for all noise levels. The automatic tuning of *h* adapts the smoothing to the noise level.

**Figure 8:**
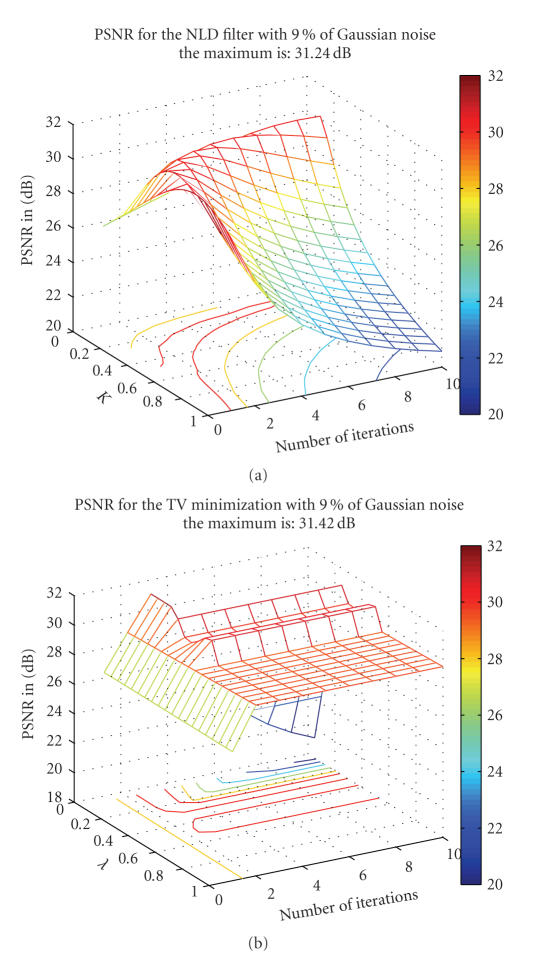
Result for the NLD filter and the TV minimization on phantom images with Gaussian noise at 9%. For the NLD filter, *K* varied from 0.05 to 1 with a step of 0.05 and the number of iterations varied from 1 to 10. For the TV minimization, *λ* varied from 0.01 to 1 with a step of 0.01 and the number of iterations varied from 1 to 10.

For NLD filter, the parameter *K* varied from 0.05 to 1 with a step of 0.05 and the number of iterations varied from 1 to 10. For TV minimization, the parameter *λ* varied from 0.01 to 1 with a step of 0.01 and the number of iterations varied from 1 to 10. The results obtained for a 9% of Gaussian noise are presented in Figure 8, but this screening was performed for the four levels of noise. It is important to underline that the results giving the best PSNR are used, but these results do not necessarily give the best visual output. Actually, the best PSNR value for the NLD filter and TV minimization are obtained for a visually under-smoothed image since these methods tend to spoil the edges. This is explained by the fact that the optimal PSNR is obtained when a good tradeoff is reached between edge preserving and noise removing.

#### 4.3.1. Quantitative results

**Figure 9:**
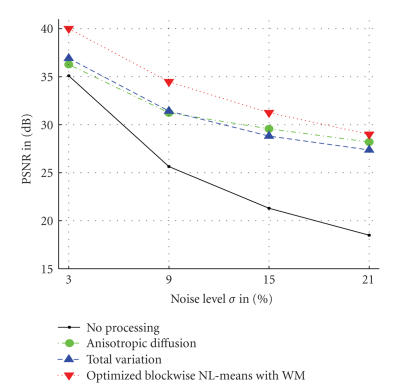
*Comparison between nonlinear diffusion, total variation, and optimized blockwise NL-means with wavelet mixing denoising.* The PSNR experiments show that the *optimized blockwise NL-means with wavelet mixing* filter significantly outperforms the well-established total variation minimization 𝔹 ^5^ process and the nonlinear diffusion approach.

As presented in Figure 9, our block-optimized NL-means with wavelet mixing filter produced the best PSNR values whatever the noise level is. On average, a gain of 2.15 dB is achieved compared to TV minimization and AD filter. The PSNR value between the noisy image and the ground truth is called “No processing” and is used as a reference.

#### 4.3.2. Visual assessment

**Figure 10:**
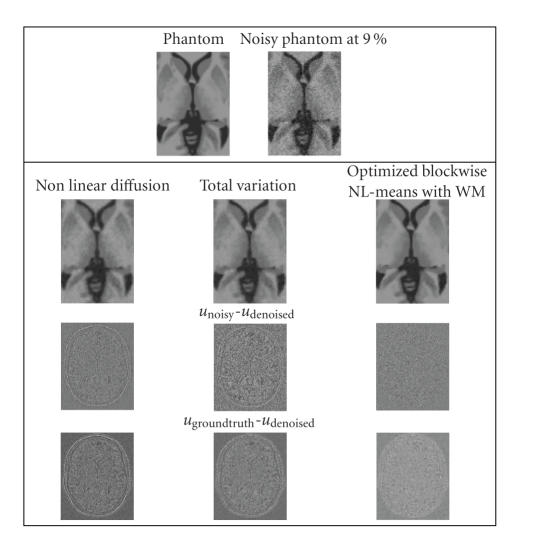
*Comparison between nonlinear diffusion, total variation, and our optimized blockwise NL-means with wavelet mixing denoising on synthetic T1-w images.* Top. Zooms on T1-w BrainWeb images. Left. The “ground truth”. Right. The noisy images with 9% of Gaussian noise. Middle. The results of restoration obtained with the different methods and the images of the removed noise (i.e., the difference (centered on 128) between the noisy image and the denoised image. Bottom. The difference (centered on 128) between the denoised image and the ground truth. Left. Nonlinear diffusion denoising. Left. Nonlinear diffusion denoising. Middle. Total variation minimization process. Right. *Optimized Blockwise NL-means with WM* filter. The NL-means-based restoration better preserves the anatomical structure in the image while efficiently removing the noise as it can be seen in the image of removed noise.

Figure 10 shows the denoising results obtained by the NLD filter, the TV minimization, and our *optimized blockwise NLM with WM*. Visually, the NL-means-based approach produced the best denoising. The removed noise (see middle of Figure 10) shows that the proposed method removes significantly less structures than NLD filter or TV minimization. Finally, the comparison with the “ground truth” underlines that the NL-means restoration gives a result very close to the “ground truth” and better preserves the anatomical structure compared to NLD filter and TV minimization.

## 5. EXPERIMENTS ON CLINICAL DATA

**Figure 11:**
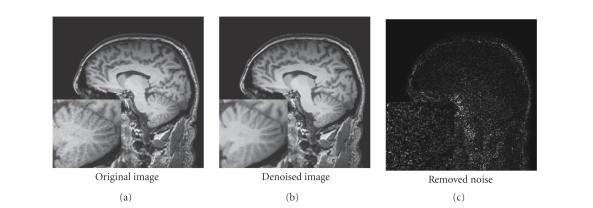
Fully automatic restoration obtained with the optimized blockwise NL-means with wavelet mixing filter on a 3 Tesla T1-w MRI data of 256^3^ voxels in less than 4 minutes on a DualCore Intel(R) Pentium(R) D CPU 3.40 GHz.

The T1-weighted MR images used for experiments were obtained with T1 sense 3D sequence on 3T Philips Gyroscan scanner. The restoration results, presented in Figure 11, show good preservation of the cerebellum. Fully automatic segmentation and quantitative analysis of such structures are still a challenge, and improving restoration schemes could greatly improve these processings.

## 6. DISCUSSION AND CONCLUSION

This paper presented a fully automated blockwise version of the nonlocal means filter with subbands wavelet mixing. Experiments were carried out on the BrainWeb dataset [26] and real dataset. The results on phantom shows that the proposed optimized blockwise NL-means with subbands wavelet mixing filter outperforms the classical implementation of the NL-means filter and the optimized implementation presented in [21, 22], in terms of PSNR values and computational time. Compared to the classical NL-means filter, our implementation (with block selection, blockwise implementation, and wavelet subbands mixing) considerably decreases the required computational time (up to a factor of 20) and significantly increases the PSNR of the denoised image. The comparison of the filtering process with and without wavelet mixing shows that the subbands mixing better preserves edges and better enhances the contrast between the tissues. This multiresolution approach allows to adapt the smoothing parameters along the frequencies by combining several denoised images. The comparison with well-established methods such as NLD filter and TV minimization shows that the NL-means-based restoration produces better results. Finally, the impact of the proposed multiresolution approach based on wavelet subbands mixing should be investigated further, for instance, when combined to the nonlinear diffusion filter [4] and the total variation minimization [3].

## Figures and Tables

**Table 1:** Comparison of different implementations of NL-means in terms of computational time and denoising quality. The computational time was obtained with multithreading on a DualCore Intel(R) Pentium(R) D CPU 3.40 GHz. These results were obtained on a T1-w phantom image of 181 × 217 × 181 voxels with 9% of noise.

	Computational time (s)	PSNR (dB)
NLM	4208	32.59
Blockwise NLM	734	31.73
Optimized NLM	778	34.44
Optimized blockwise NLM	135	33.75
Optimized blockwise NLM with WM	181	34.47
